# Paternal Radiofrequency Electromagnetic Radiation Exposure Causes Sex-Specific Differences in Body Weight Trajectory and Glucose Metabolism in Offspring Mice

**DOI:** 10.3389/fpubh.2022.872198

**Published:** 2022-05-06

**Authors:** Song Yan, Ying Ju, Jie Dong, Hui Lei, Jun Wang, Qian Xu, Yefei Ma, Jingjing Wang, Xiaohong Wang

**Affiliations:** Department of Gynecology and Obstetrics, Tangdu Hospital, Air Force Medical University, Xi'an, China

**Keywords:** RF-EMR, fertility, offspring, body weight trajectories, glucose metabolism

## Abstract

Nowadays, concerns about the harmful effects of radiofrequency electromagnetic radiation (RF-EMR) on male fertility and offspring health are growing. In the present study, we investigated the effects of long-term exposure (at least 10 weeks) to the RF-EMR [2.0 GHz; power density, 2.5 W/m^2^; whole-body specific absorption rate (SAR), 0.125–0.5 W/kg] on male mice fertility and F1 growth and glucose metabolism. No significant injuries were observed in testis organization, sperm quality, and pregnancy rate. However, mice exposed to RF-EMR exhibited a significantly elevated apoptosis rate in testis germ cells. Interestingly, paternal RF-EMR exposure resulted in sex-specific weight trajectory differences and glucose metabolism changes in male F1 mice but not in female F1 mice. The changed glucose metabolism in F1 male may result from the altered gene expression of liver Gck. These data collectively suggested that 2.0 GHz RF-EMR whole-body exposure of male mice does not cause obvious impairment in testis, sperm quality, and pregnancy rate. Paternal RF-EMR exposure causes male-specific alterations in body weight trajectories and glucose metabolism of F1.

## Introduction

Nowadays, non-ionizing radiofrequency electromagnetic radiation (RF-EMR) is ubiquitous, especially in the industry, military, radio, and wireless communications sectors. The potential adverse effects of RF-EMR on human health and offspring development have obtained lots of public attention (1–4). Notably, the International Agency for Research on Cancer (IARC) has categorized RF-EMR as possible carcinogens to humans (5, 6).

As the testis is reported to be one of the most sensitive organs to RF-EMR, it is essential to evaluate the effect of RF-EMR on male fertility. Sperms also play critical roles in offspring health, as they could carry some paternal epigenetic information to the embryo (7, 8). Many investigations have been conducted on the effect of RF-EMR on sperm quality, but the results are inconsistent (9–11). A recent systematic review and meta-analysis including 39 investigations presented that the pooled results of human cross-sectional studies did not support an association between mobile phone use and sperm quality decline. However, pooled results of animal studies indicated that mobile phone RF-EMR exposure could suppress sperm motility and viability (12). Furthermore, most previous studies were focused on sperm quality; more crucial issues like whether the RF-EMR impacts male fecundity remains unclear (12, 13).

Epidemiological studies have reported that paternal occupational exposure to radiofrequency electromagnetic fields is associated with adverse pregnancy outcomes (such as preterm birth) and congenital disabilities (14, 15). In addition, a decreased birth weight of F1 from paternal RF-EMR exposed rats has been reported in animal experiments (16, 17). Glucose metabolism plays a crucial role in the regulation of body weight. Dysregulation of glucose metabolism is a risk factor for many diseases, including diabetes, obesity, adverse cardiovascular events, etc. (18, 19). However, the effect of paternal RF-EMR exposure on offspring glucose metabolism is unknown.

In this study, we aimed to evaluate the effects of RF-EMR exposure on the testis injury, sperm quality, male fecundity in mouse models, and to further explore whether offspring glucose metabolism is altered, which may provide more knowledge on possible effects of RF-EMR exposure on human health.

## Materials and Methods

### Animals and Study Design

Male specific-pathogen-free (SPF) C57BL/6 J mice (6–8 weeks old) were purchased from the Experimental Animal Center, Air Force Medical University (Xi'an, China). They acclimated for −7 days with tap water and a pelleted basal diet before the start of the experiments. All animal experiments were performed according to the Animal Protection Guidelines of Air Force Medical University (SYXK2019-001).

**Figure 1:**
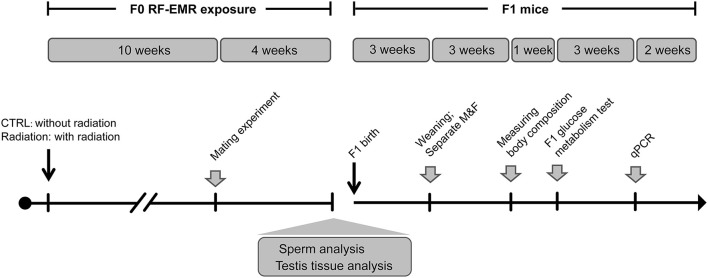
Experiment design of longitudinal mouse exposure study. RF-EMR, radiofrequency electromagnetic radiation. CTRL, mice in sham group; Radiation, mice in RF-EMR exposure group; M, male F1 mice; F, female F1 mice.

To test the impact of RF-EMR on male fertility, C57BL/6 J mice (*n* = 8 in each group) were randomly assigned to CTRL (sham exposure group) or Radiation group (RF-EMR exposure) (Figure 1). After 10 weeks of exposure, male mice were housed with one female mouse overnight to test fecundity. This mating experiment was conducted once in a week for four consecutive times. The vaginal plugs were checked, and the mice were separated the next morning once the copulation had taken place. Litter sizes and male pups were recorded on delivery. After mating, the Radiation group mice were again subject to radiation. Sperm and testis were collected for further analysis when mating experiments were done. Thus, it was 14 weeks of radiation when we analyzed testis organization and sperm quality (Figure 1).

Furthermore, offspring from CTRL or Radiation mice were raised to explore the effects of paternal RF-EMR exposure on F1 development and glucose metabolism. Pups were numbered per the order of weighing on the postnatal day 1 and culled to 6–8/litter on the postnatal day 3 (maximally balancing the sex ratio). After weaning, the No.1 and No.2 male and female F1 in each litter were weighed weekly to monitor the growth trajectory until 12-week-old (Figure 1). In addition, postnatal 6-week-old No.1 and No.2 male F1 in each litter were picked to analyze body composition as described below. Then, at 7 weeks of birth, the No.1 and No.2 male and female F1 in each litter were chosen to perform glucose metabolism analysis. After all these metabolic tests were done, F1 mice were euthanized, and their livers were harvested at 10 weeks of age for gene expression analysis (Figure 1).

### Radiofrequency Electromagnetic Radiation Exposure

As illustrated in Supplementary Figure 1, the in-house 2.0 GHz RF-EMR exposure system was comprised of a CW signal generator (f = 2.0 GHz, power = −22.0 dBm; 1441A, China Electronics Technology Instruments Co., Ltd., China), an amplifier (output powers = 8.4 W; SWSPA-C200S/C-2, Chengdu SWIEE Power Electronics Technology Co., Ltd, China), and a horn antenna (8.94 dB gain, CW; LB-1080, A-INFO, China). The distance between the antenna and the target animals was 1.3 m, ensuring that the mice were in the far-field of the antenna.

The power density in the point of mice cage was 2.5 W/m^2^, as measured by an electromagnetic fields meter (PMM8053A, PMM Costruzioni Electtroniche Centro Misure Radio Electriche S.r.l., Milan, Italy). The C57BL/6 J mice in the Radiation group were placed freely in a plexi glass cage (one mouse per cage), allowing for the transmission of electromagnetic waves. Mice in the CTRL group were raised in similar cages without the exposure of RF-EMR. As indicated, mice were exposed to 2.0 GHz frequency, 3 h/day (9:00 a.m. to 12:00 a.m.) RF-EMR. The animals had free access to food and water when they were exposed. Since the incidence and polarization of free movement mice kept changing during exposure, the estimated whole-body specific absorption rate (SAR) value ranged from 0.125 to 0.5 W/kg, which were calculated with the help of a prolate spheroidal model of a medium mouse (20–22).

### Sperm Quality

For sperm quality analysis, cauda epididymis was cut into pieces by placing in the Human Tubal Fluid (HTF) Medium (FUJIFILM) for sperm swim up at 37°C for 30 min. Hemocytometer was used for sperm count. The sperm suspension was also detected by flow cytometry (FACS Aria II, BD Biosciences) to detect sperm apoptosis and viability via Annexin-V/7-AAD staining (Biolegend). For reactive oxygen species (ROS) detection, the sperm was washed with PBS and incubated with 10 μMDCFH-DA (Beyotime, China) in PBS for 30 min at 37°C. After incubation, samples were washed again and detected by flow cytometry.

### Immunofluorescence

For BrdU staining, mice were intraperitoneally injected with BrdU (Sigma, B5002, 50 mg/kg) 4 h before testis harvesting. Paraffin sections (3 μm) were deparaffinized in xylol and rehydrated. Antigen retrieval was performed by heating in citric acid buffer (pH = 6.0) at 95°C for 15 min. Then slides were incubated with 4 M HCl at room temperature for 30 min, and washed thrice with 1.5 M Tris-HCl (pH = 8.8). Slides were incubated with primary antibodies (BrdU, Santa Cruz, sc-32323; 1:100) overnight at 4°C, and subsequently incubated with Alexa Fluor 488 anti-mouse secondary antibody (Thermo Scientific, A11001, 1:500). Nuclei were stained with DAPI and tissues were analyzed by a confocal microscope (Nikon, Japan).

### TUNEL Staining

Sections were prepared and rehydrated for TUNEL staining according to the instructions (Beyotime, China). Briefly, sections were firstly incubated with proteinase K (20 μg/ml) for 15 min at 37°C followed by washing, then incubated with 50 μL TUNEL mix for 60 min at 37°C, and then were incubated with DAPI for 10 min at room temperature.

### Blood-Testis Integrity Assay

The permeability of the blood-testis barrier was assessed using Inulin-FITC (Sigma, F3272). Briefly, a total of 20 μL inulin-FITC (10 mg/mL in PBS) was injected into the interstitium of the testes. The animals were euthanized 40 min later, and the testes were immediately collected and embedded in optimal cutting temperature (OCT) tissue freezing medium (Leica). Nuclei were stained with DAPI, and fluorescence images of the cryosections were captured with a fluorescence microscope (Olympus).

### Evaluation of Male Fertility

After 10 weeks of radiation, each male mouse was housed with one 8-week-old female C57BL/6 J mouse (in proestrus or estrus) overnight inside a well-ventilated room kept at 24°C with a 12-h light-dark cycle. The female mice were checked for vaginal plugs and separated, if copulation had taken place. Litter sizes and pup numbers (for all mice) were recorded on delivery.

### Body Weight and Body Composition Measurement

All the newborns' weights were measured at the age of 1 day. After weaning at 3 weeks of age, we weighed some of the offspring every week from 3 to 12 weeks of age. At 6 weeks of age, the body composition of F1 male mice was measured to obtain lean mass and fat mass by nuclear magnetic resonance (NMR) by the EchoMRI-500 (USA).

### Metabolic Studies

For all experiments, mice blood glucose was determined with a blood glucose meter (YUWELL, China) from the tail vein blood. In brief, mice were given free access to the food before measuring fed glucose at 4 pm. Fasting blood glucose levels were measured at 8 am after 12 h fasting. Both fed and fasting blood glucose were measured at 7 weeks of age for F1 offspring.

The intraperitoneal glucose tolerance test (IPGTT) and pyruvate tolerance test (PTT) were performed at 9 am, after 12 hrs fasting. In contrast, the insulin tolerance test (ITT) was performed at 2 pm, following a 6 hrs fasting. Glucose (1 g/kg, Sigma), pyruvate (1.5 g/kg, Sigma), or insulin (0.75 IU/kg, Novo Nordisk, Denmark) was administrated intraperitoneally to the subject. The glucose level from the tail vein blood was measured at 0, 15-, 30-, 60-, and 120-mins post injection. IPGTT, PTT, and ITT were performed in F1 mice at 7-, 8- and 9- weeks of age, respectively.

### Quantitative Real-Time Polymerase Chain Reaction

Total RNA was extracted with RNAiso Plus Reagent (TaKaRa, Japan) and then reverse-transcribed by Prime Script RT reagent kit (TaKaRa, Japan). Quantitative PCR was performed using TB Green (TaKaRa) on a Real-time PCR Detection System (Bio-Rad, United States). Cycle threshold (CT) value was used to calculate the fold change by the 2^−ΔΔCT^ method, and the relative mRNA expression was normalized to Actin. The primer sequences of Gck, Pklr, G6pc, Pck1, Pygl, Foxo1, Irs1, Irs2, and Actin are listed in Supplementary Table 1.

### Statistics

All experiments were performed at least three times, data are showed as means ± standard error of the mean (SEM), and statistical analyses were carried out using GraphPad Prism 7. The area under the curves (AUCs) of glucose levels in IPGTT, ITT, and PTT were calculated with GraphPad Prism 7. Normality of data was tested first, and then a two-tailed Student's *t*-test was used to test differences between samples. *p* < 0.05 was considered as significant.

## Results

### Impact of RF-EMR Exposure on Mice Testis

**Figure 2:**
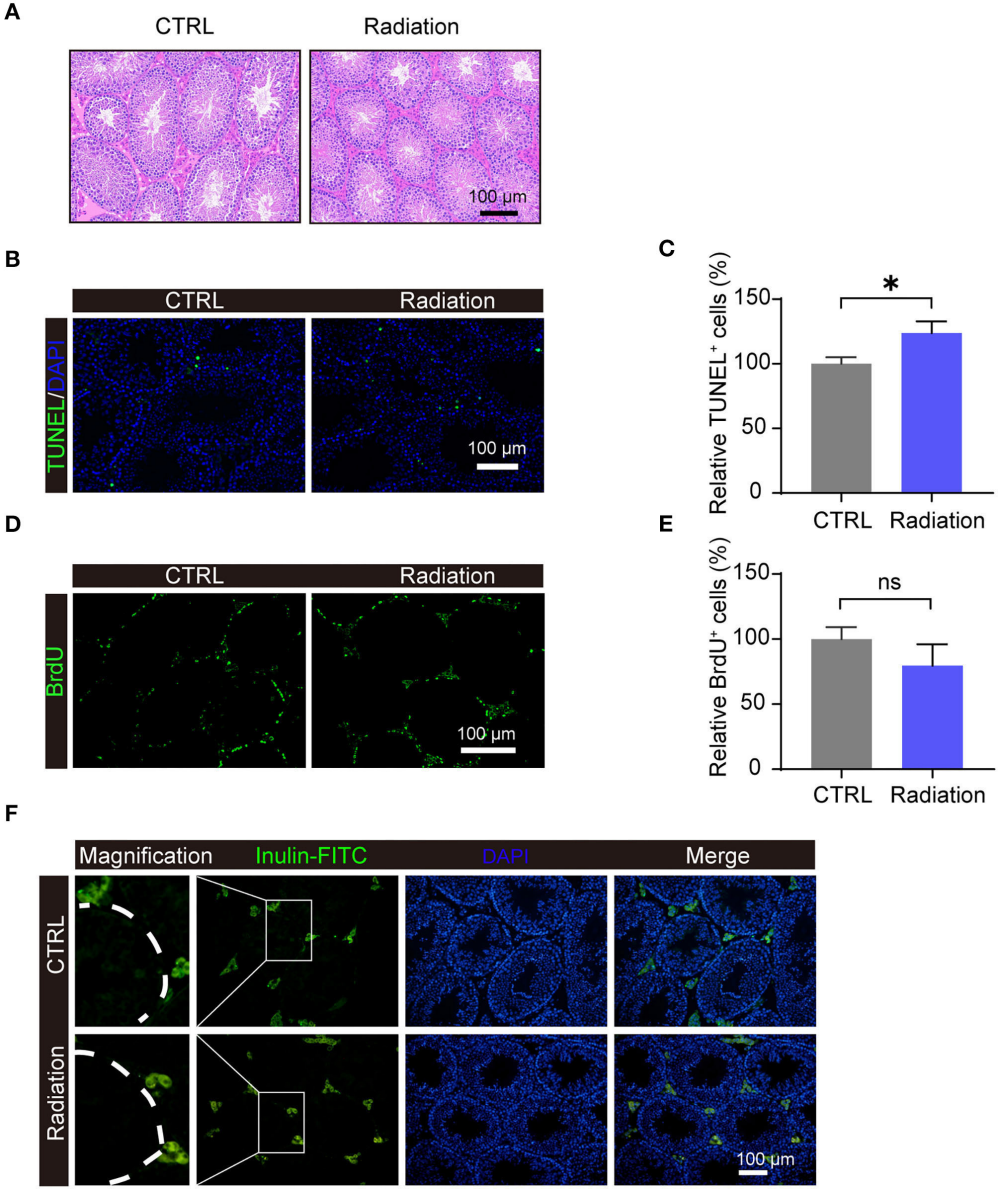
Impact of RF-EMR (electromagnetic radiation) exposure on murine testis. **(A)** H&E staining of the testes from CTRL or Radiation mice. **(B,C)** Detection of apoptosis in testes of CTRL and Radiation mice using TUNEL staining. **(D,E)** Detection of BrdU incorporation post intraperitoneal injection in the testis of CTRL and Radiation mice. **(F)** In the testis of both CTRL and Radiation group, Inulin-FITC was located near the base of the seminiferous tubule. **(A–F)** CTRL, mice in sham group; Radiation, mice in RF-EMR exposure group. *P < 0.5; ns, not significant. *n* = 6 for both groups. The Student's *t*-test was performed to compare groups. Scale bar = 100 μm.

After 14 weeks of exposure, mice testis in the Radiation group (RF-EMR exposure) presented no morphology difference compared with the CTRL group (without exposure) by H&E staining (Figure 2A). Apoptosis of testis germ cells was significantly induced in the Radiation group as determined by TUNEL assay, while the proliferation rate detected by BrdU staining was not significantly influenced (Figures 2B–E). The blood-testis barrier plays a pivotal role in male fertility (23); we observed its integrity by Inulin-FITC injection (Figure 2F). The fluorescent image indicated that Inulin-FITC distribution was restricted to the testis interstitial space in both groups, suggesting that the murine blood-testis barrier might not be affected after 14 weeks of RF-EMR exposure. Collectively, these data indicated that testis germ cells apoptosis is induced after RF-EMR exposure in our experimental conditions.

### Effects of RF-EMR Exposure on Sperm Quality

**Figure 3:**
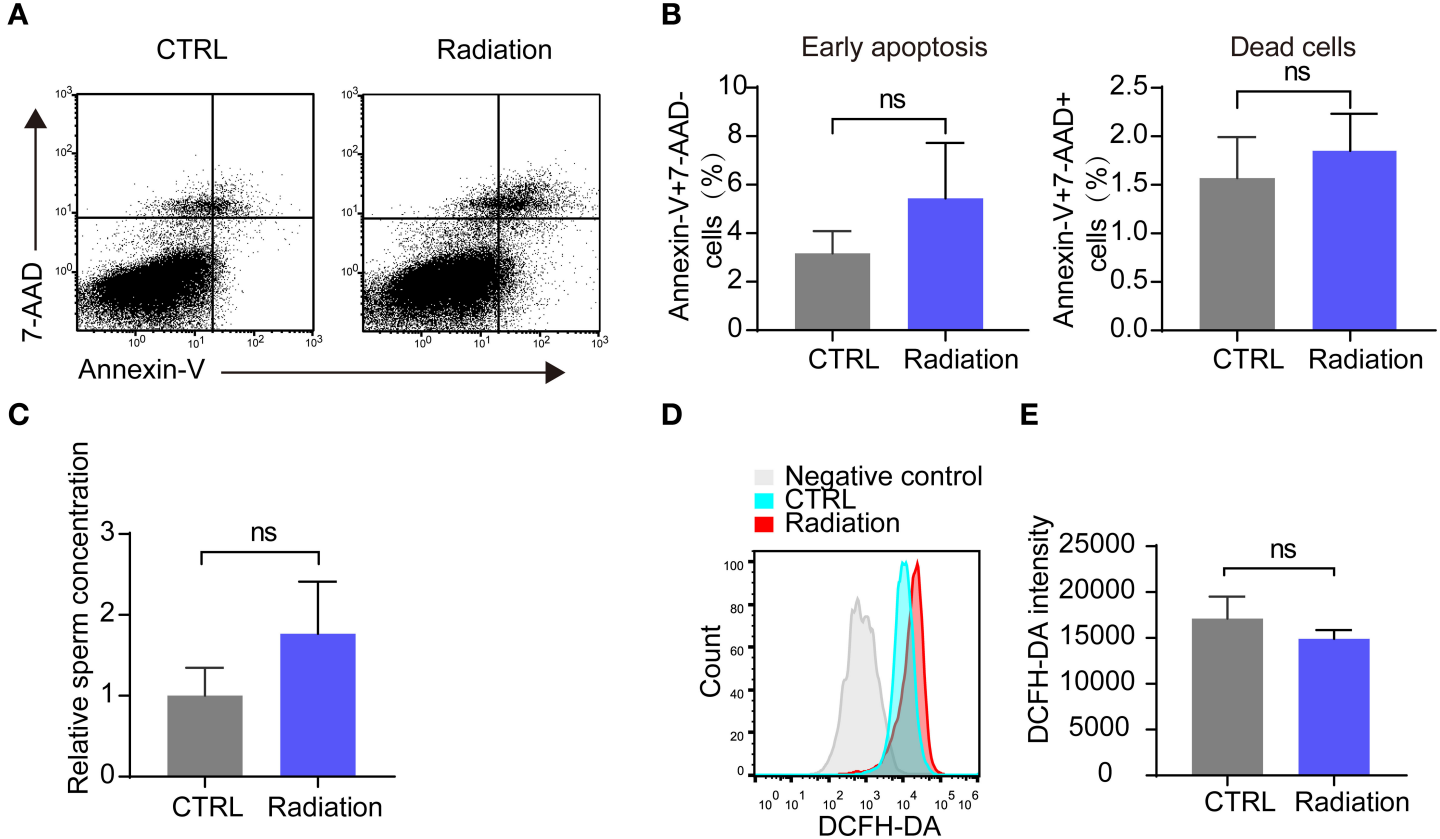
Effects of RF-EMR on sperm quality. **(A,B)** The sperm apoptosis in the epididymis of Radiation and CTRL mice were detected by flow cytometry. **(C)** Epididymissperm numberof both groups determined by haemocytometer. **(D,E)** Mice sperm ROS (reactive oxygen species) were tested by DCFH-DA on a flow cytometry. **(A–E)** CTRL, mice in sham group; Radiation, mice in RF-EMR exposure group. ns, not significant. *n* = 6 for both groups. Student's *t*-test.

We then analyzed the influence of RF-EMR exposure on murine sperm quality. Early apoptosis and death rate of sperm in the radiation mice group showed no significant difference compared with that of the control group, as presented by Annex in-V/7-AAD staining (Figures 3A,B). Epididymis sperm concentrations were also not changed (Figure 3C). Furthermore, there was no difference in sperm ROS between the two groups (Figures 3D,E). These data revealed that RF-EMR exposure in our experimental conditions does not influence sperm quality.

### Influence of RF-EMR Exposure on Male Mice Fecundity

**Figure 4:**
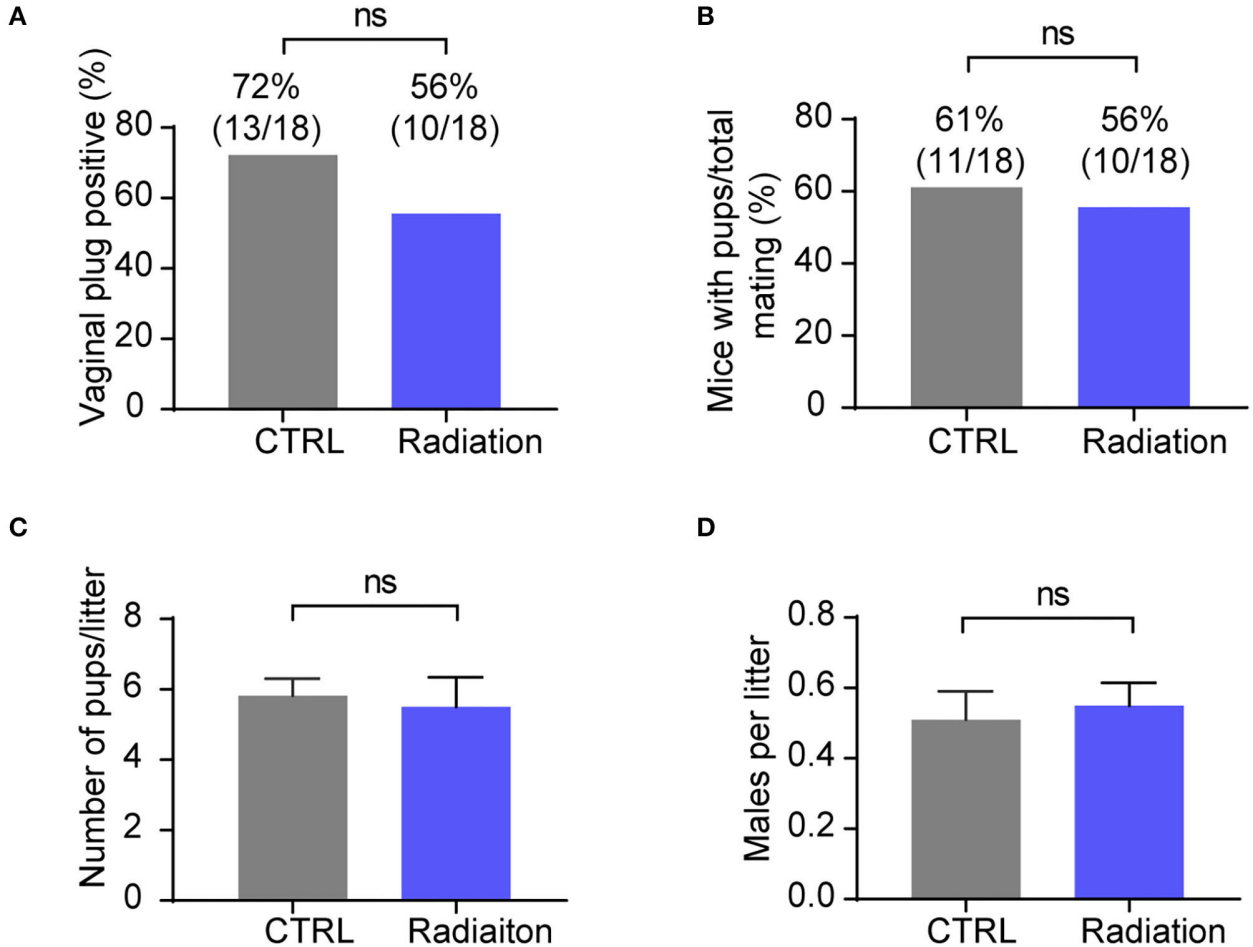
RF-EMR exposure does not influence male mice fertility. **(A,B)** Male fertility reflected by female mice pregnancy rate. Chi-square test. **(C)** Average litter sizes of CTRL or Radiation mice offspring. **(D)** Sex ratio of pups from CTRL or Radiation mice. **(A–D)** CTRL, mice in sham group; Radiation, mice in RF-EMR exposure group. ns, not significant. **(C,D)** *n* = 8–11. A two-tailed Student's *t*-test was performed.

We further investigated the effect of RF-EMR exposure on mice fertility by detecting the female mice pregnancy rate and sex ratio of pups. The results showed that RF-EMR exposure did not influence male mice fertility and had no effects on litter size and sex ratio (Figures 4A–D). These data underscored that our experiment's RF-EMR exposure does not affect male mice fertility.

### F0 Glucose Metabolism Is Not Affected by RF-EMR

To assess the effects of RF-EMR on glucose metabolic homeostasis in male mice, we measured the body weight, the blood glucose level, and the glucose tolerance (IPGTT) in mice following a period of 14 weeks RF-EMR. There was no difference in body weight between the CTRL and the Radiation mice (Supplementary Figure 2A). Neither fasting nor fed blood glucose was altered in the F0 exposed male mice (Supplementary Figure 2B). Also, the glucose tolerance was not influenced (Supplementary Figure 2C). These results demonstrated that RF-EMR does not impact F0 mice glucose metabolism.

### Paternal RF-EMR Exposure Causes Sex-Specific Differences in Body Weight Trajectories

**Figure 5:**
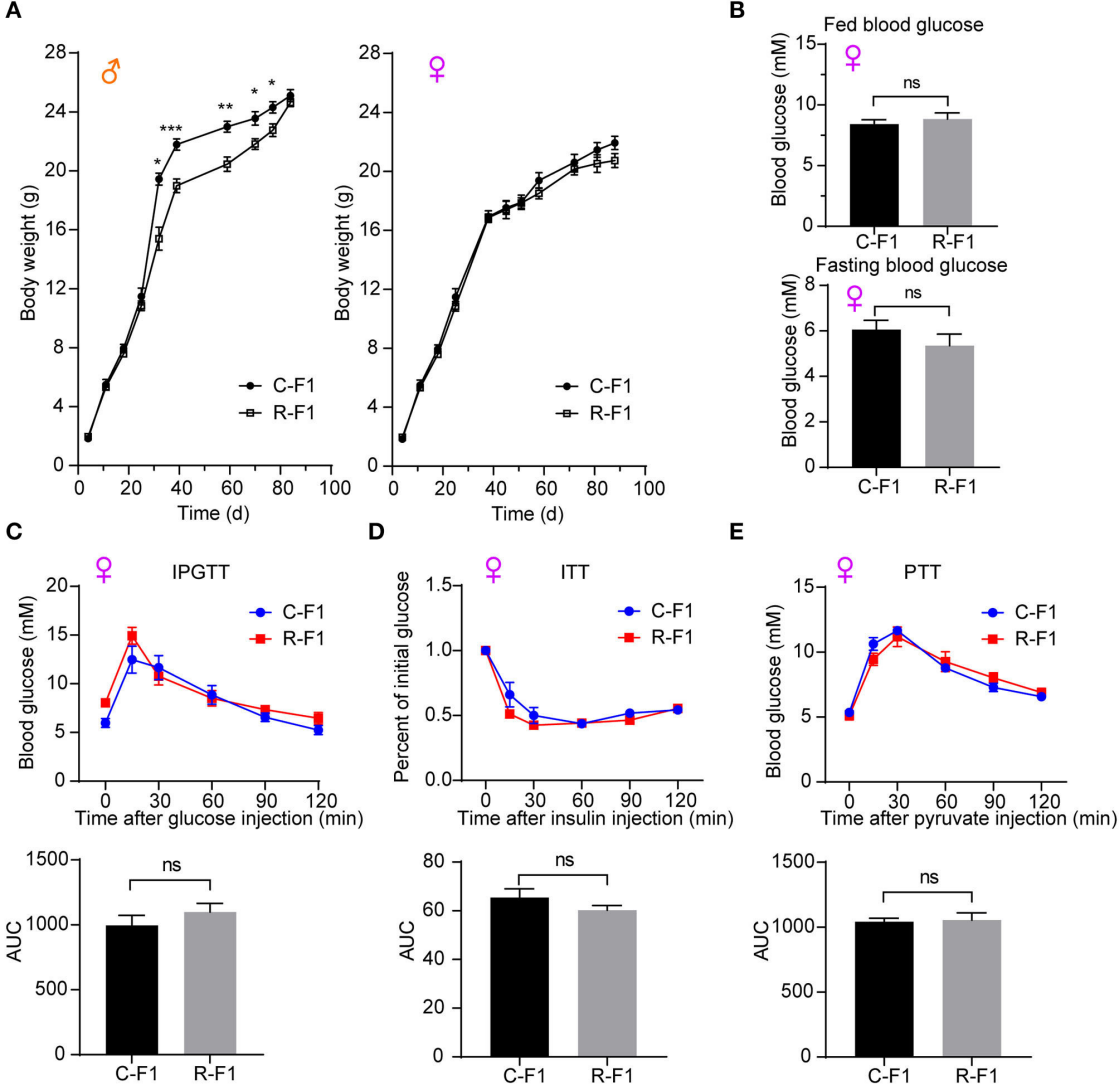
Metabolic phenotypes in female offspring of exposed fathers. **(A)** The growth curves of F1 mice. **(B)** The levels of fed blood glucose and fasting glucose in the two female F1 group mice at 7 weeks of age. **(C–E)** The intraperitoneal glucose tolerance test (IPGTT, 7 weeks of age), insulin tolerance test (ITT, 8 weeks of age), the pyruvate tolerance test (PTT, 9 weeks of age), and the area under the curve (AUC) in the female F1 mice. **(A–E)** C-F1, F1of the control father; R-F1, F1 of the Radiation father; ns, not significant; *p < 0.05, **p < 0.01, ***p < 0.001. Student's *t*-test. **(A)** *n* = 9–10. **(B–E)** *n* = 6.

As Figure 5A indicated, there was no difference in birth weights of F1 between the CTRL father (C-F1) group and the Radiation father (R-F1) group. Interestingly, the weights of male offspring but not female offspring remained significantly lower in the R-F1 group than those in the C-F1 group after weaning. However, no significant difference was detected after 12 weeks of age between the R-F1 and C-F1 groups (Figure 5A). These results suggested that the body weight trajectories of F1 males are influenced during the period of adolescence while that of females are not.

### Female F1 Glucose Metabolism Is Not Altered

To explore the potential effects of paternal RF-EMR exposure on the female offspring's metabolism, the blood glucose level and the glucose homeostasis (evaluated by IPGTT, ITT, and the PTT) in female F1 mice were investigated. There was no difference in parameters as aforementioned between female C-F1 and R-F1 groups (Figures 5B–E). These results underscored that paternal RF-EMR exposure does not impact F1 female mice glucose metabolism.

### Male F1 Glucose Metabolic Parameters of Exposed Father Are Influenced

**Figure 6:**
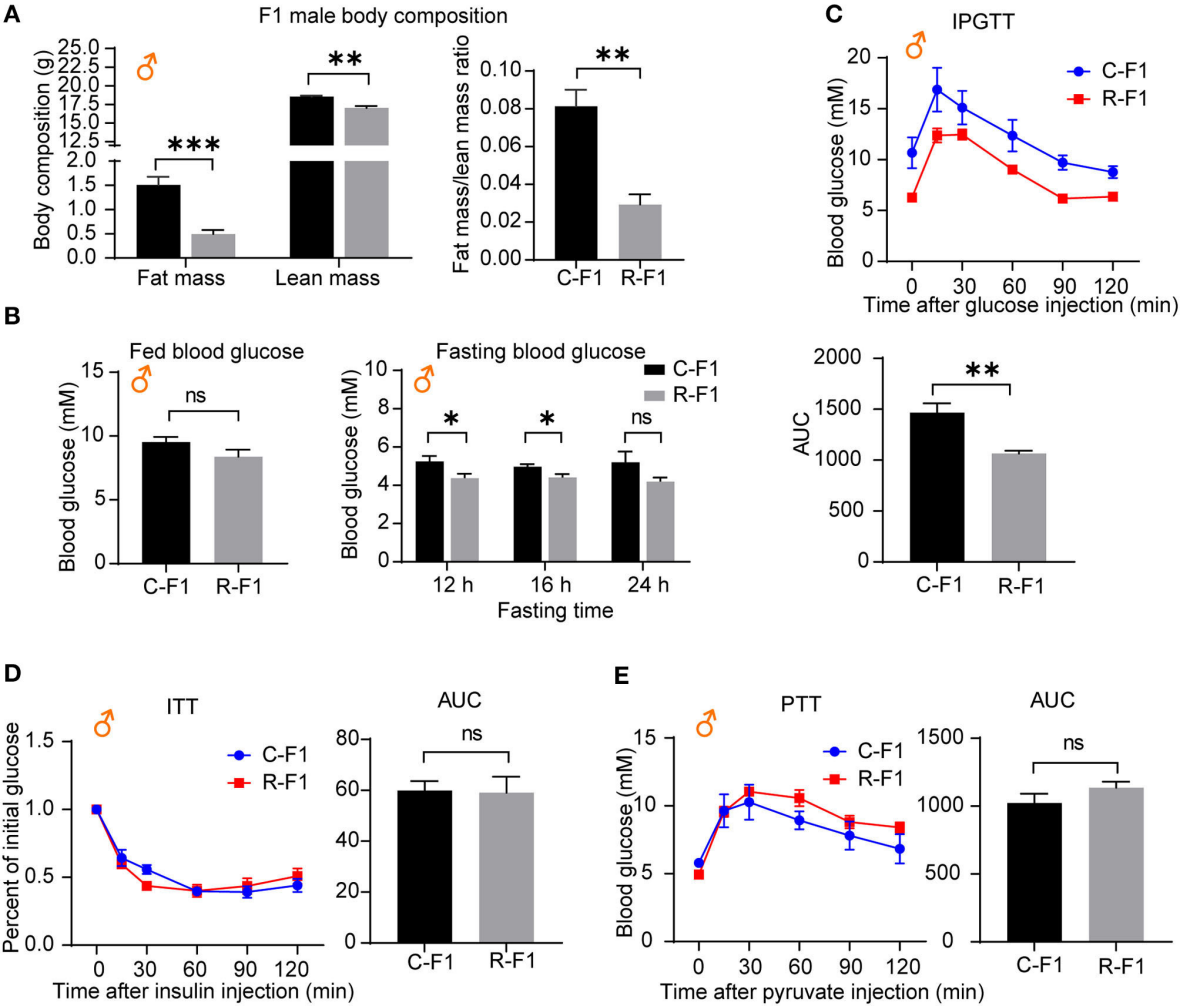
Metabolic profiles in male offspring from exposed fathers are influenced. **(A)** The body compositions of male F1 mice 45 days after birth. **(B)** The levels of fed blood glucose and fasting glucose in male F1 mice (7 weeks of age). **(C–E)** The intraperitoneal glucose tolerance test (IPGTT, 7 weeks of age), insulin tolerance test (ITT, 8 weeks of age), the pyruvate tolerance test (PTT, 9 weeks of age), and the area under the curve (AUC) in the male F1 mice. ns, not significant. **(A–E)** C-F1, F1 of the control father; R-F1, F1 of the Radiation father. *p < 0.05, **p < 0.01, ***p < 0.001. *n* = 6 for both groups. A two-tailed Student's *t*-test was performed.

As an altered growth curve was observed in the male F1 mice, we next determined the body composition of F1 mice, and the data indicated that R-F1 male mice gained significantly less fat mass than C-F1 (Figure 6A, 45 days of age). Furthermore, the fasting blood glucose level and the AUC (the area under the curve) of IPGTT were significantly lower than that of C-F1 (7 weeks of age), while the AUC of ITT (8 weeks of age)and PTT (9 weeks of age) did not change (Figures 6B–E).

**Figure 7:**
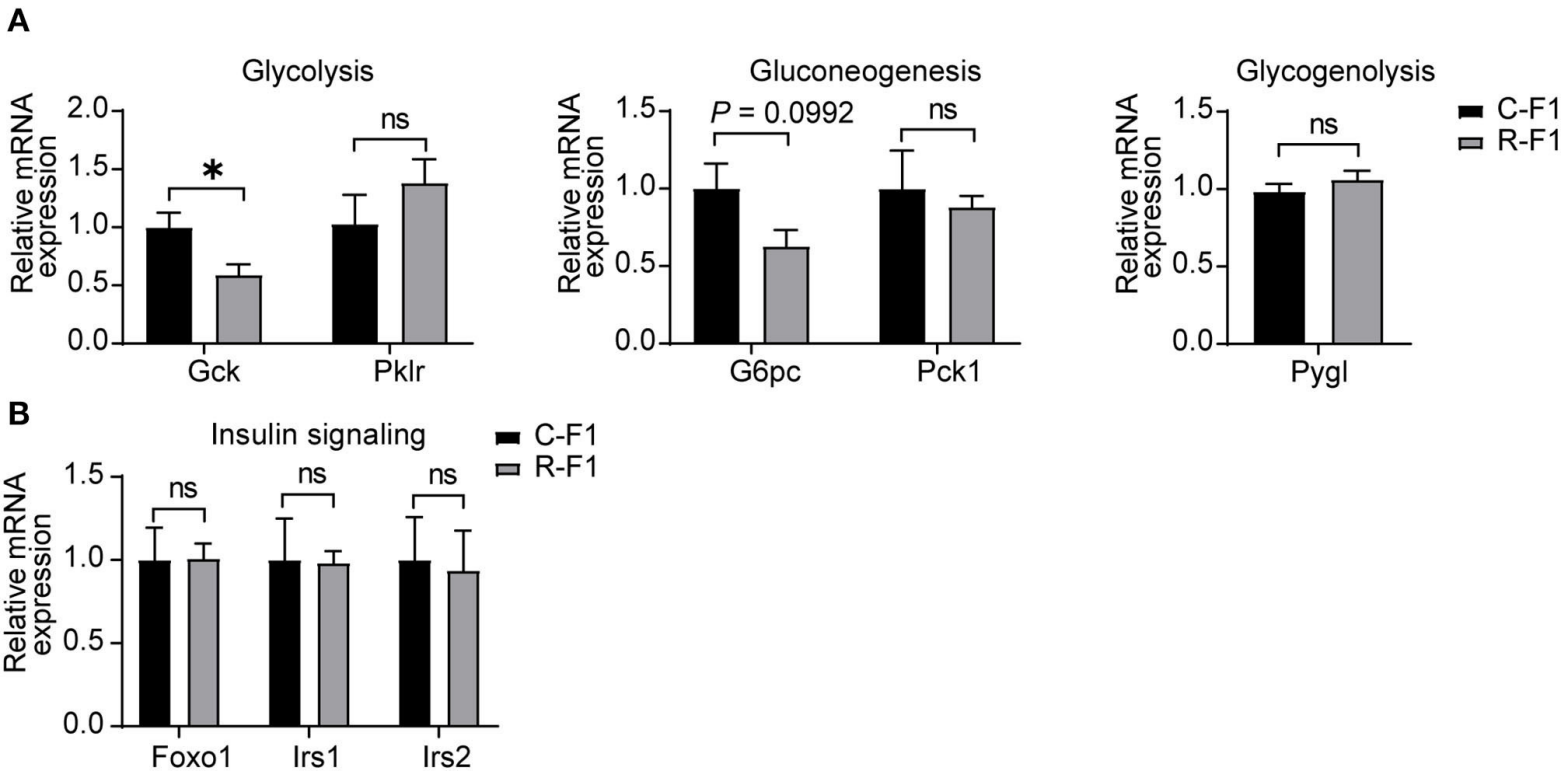
Disturbance ofliver glucose metabolism-associated gene expression in the male F1 mice of the exposed father. **(A)** Relative mRNA levels of glycolytic (Gck and Pklr), gluconeogenic genes (G6pc and Pck1), and glycogenolytic gene (Pygl) in livers of each group. **(B)** Gene expression of insulin signaling between male F1 mice. ns, not significant. **(A,B)** C-F1, F1of the control father; R-F1, F1 of the Radiation father. *p < 0.05. *n* = 6 for each group. Student's *t*-test.

Hepatic glucose production plays an essential role in regulating glucose homeostasis through glycolysis, gluconeogenesis, and glycogenolysis (24, 25). To examine the underlining mechanism of male R-F1 glucose metabolism alteration, next, we focused on glucose metabolism associated enzymes of the liver. As shown in Figure 7A, of these key enzymes, the mRNA level of glycolytic enzyme Gck was significantly down regulated, while the other enzymes were not influenced. In addition, insulin signaling, which is essential to the regulation of live glucose metabolism, was not altered (Figure 7B).

## Discussion

Nowadays, people are increasingly worried about the adverse effects of non-ionizing radiofrequency electromagnetic radiation (RF-EMR) on male reproduction and offspring health (26, 27). Previous human or animal experiments were mainly focused on the effects of RF-EMR radiation on sperm quality (sperm count, motility, viability, morphology, etc.), and the conclusions are controversial (28, 29). Additionally, there is a paucity of literature exploring the potential effects of RF-EMR on male fecundity and offspring health (30). The International Commission on Non-Ionizing Radiation Protection (ICNIRP) sets the limit of whole-body exposure in the general population at 0.08 and 0.4 W/kg for the occupational population (31). In our experiment, we set up an RF-EMR system with a frequency of 2.0 GHz, power intensity of 2.5 W/m^2^, and the estimated whole-body specific absorption rate (SAR) value ranged from 0.125 W/kg to 0.5 W/kg. After long-term exposure [>10 weeks, which is about two complete spermatogenic cycles; note that the overall differentiation process from spermatogonia to mature spermatozoa requires ~35 days in mice (32)], no significant injuries in testis organization, sperm quality, and pregnancy rate was showed, although a significantly elevated testis germ cells apoptosis was found. Furthermore, paternal RF-EMR exposure causes male-specific differences in body weight trajectories, and the glucose metabolism is altered in adolescent F1 males but not in female F1 mice from RF-EMR exposed fathers.

Regarding the injuries of RF-EMR exposure on testis and sperm, the results remain debatable. Kesari and Behari reported that mobile phone RF-EMR radiation for 2 h per day for 45 days in the rat could lead to sperm morphology alteration and a decrease in testosterone level (16). Furthermore, a recent study concluded that 4G cellphone RF-EMR could directly cause injury of the testis, disorder of the blood-testis barrier, andreduction of semen qualityin the rat (33). However, another investigation also reported that C57BL/6 mice exposed to 1.5 GHz high-power microwavesdid not cause noticeabledamage to the reproductive system (including sperm quality) (34). Our study found no change in the percentage of sperm apoptosis and dead sperm cells in the RF-EMR group after exposing mice for 14 weeks. Factors like different species used in experiments, exposure devices, and exposure time might contribute to the heterogeneity of animal studies (12, 13).

When studying the effects of RF-EMR on fertility of male mice, only evaluating sperm quality is not enough; elucidating possible effects of RF-EMR exposure on a male's ability to have offspring by mating experiment might be more important (35). However, these kind of studies are rare (30). Our investigation found that RF-EMR exposure did not impact the pregnancy rate in our experimental conditions. Additionally, the litter size and offspring sex ratio in our experiments were not altered. A previous study performed in male rats showed that the pup weight and litter size of radiation father that suffered 45 days mobile phone RF-EMR is significantly decreased (16). In adult rats, Yu et al. also did not find significant fertility potential declination after 50 or 100 days RF-EMR exposure (17). Moreover, a period of 150 days exposure did not affect the successful mating rate (13/15 in the control group vs. 10/14 in the exposure group), either (17). Therefore, male fertility after RF-EMR exposure might not get decreased.

To the best of our knowledge, we are the first to find that paternal RF-EMR exposure causes sex-specific differences in F1 body weight trajectories. The body weight gain in the female offspring of paternal RF-EMR is similar to that of control. However, the F1 male mice growth curve from exposed father lag behind those of F1 control mice across the period of adolescence (4–12 weeks of age), and this situation is compensated at 12 weeks of age. Previous studies in rats reported a decreased birth weight of F1, but we did not find the difference in our experiments (16, 17).

The glucose metabolism of F1 was further investigated. In line with the growth curve results, no difference in glucose metabolism was found in female F1 mice. In contrast, during adolescence, male F1 mice from RF- EMR father showed glucose metabolism disturbance, which may result from the altered gene expression of liver Gck. The potential mechanism of the altered male F1 glucose metabolism will be investigated in our future work.

Despite our findings from this study, there exist several limitations. First, the male mice were not fixed during RF-EMR exposure, which might cause exposure heterogeneity among these radiated mice. Keeping the mouse motionless during radiation would have been more appropriate, and if the same effects were found, our conclusions would have been strengthened. Furthermore, the offspring development and glucose metabolism were only observed to 12 weeks of birth; whether the male F1 glucose metabolism difference will disappear like body weight does remain to be investigated.

## Conclusion

The whole-body exposure of mice to 2.0 GHz RF-EMR did not cause obvious changes in testis, sperm quality, and pregnancy rate. Furthermore, paternal RF-EMR exposure causes sex-specific differences in body weight trajectories. The glucose metabolism in adolescent male F1 mice from RF-EMR exposed fathers is altered, but not that in female F1 mice.

## Data Availability Statement

The original contributions presented in the study are included in the article/Supplementary Material, further inquiries can be directed to the corresponding author/s.

## Ethics Statement

The animal study was reviewed and approved by the Experimental Animal Center, Air Force Medical University.

## Author Contributions

XW, JD, and SY designed the study. SY, YJ, JuW, HL, YM, QX, and JiW performed the experimental work. SY and YJ wrote the manuscript. XW, JD, SY, and YJ prepared and revised the article. XW was responsible for the supervision and project administration. All authors discussed, edited, and approved the final version.

## Funding

This work was supported by the Fourth Military Medical University (2018HKZL06) and the fund of Tangdu Hospital (2020TDPY003).

## Conflict of Interest

The authors declare that the research was conducted in the absence of any commercial or financial relationships that could be construed as a potential conflict of interest.

## Publisher's Note

All claims expressed in this article are solely those of the authors and do not necessarily represent those of their affiliated organizations, or those of the publisher, the editors and the reviewers. Any product that may be evaluated in this article, or claim that may be made by its manufacturer, is not guaranteed or endorsed by the publisher.
