# Supplementary material for: Paternal Radiofrequency Electromagnetic Radiation Exposure Causes Sex-Specific Differences in Body Weight Trajectory and Glucose Metabolism in Offspring Mice
Source: Front Public Health. 2022 May 6;10:872198. doi: 10.3389/fpubh.2022.872198 (PMC9120541; doi:10.3389/fpubh.2022.872198)
Supplement: Supplementary file 1 [file Data_Sheet_1.DOCX]

Supplementary Material

Paternal radiofrequency electromagnetic radiation exposure causes sex-specific differences in body weight trajectory and glucose metabolism in offspring mice

# Supplementary Figures and Tables

## Supplementary Figures


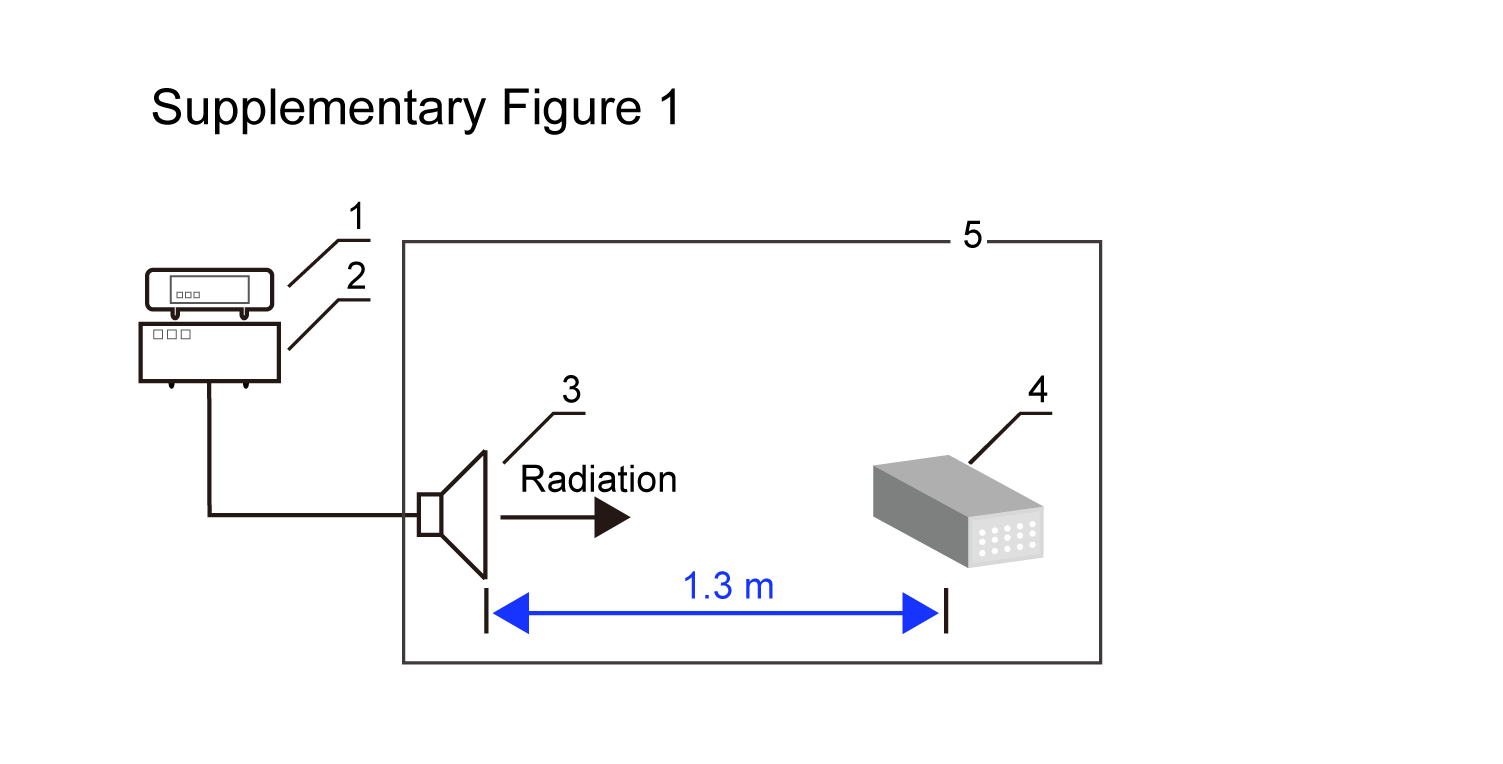


**Supplementary Figure 1.** **Illustration of the microwave radiation exposure system.** 1, radiofrequency electromagnetic radiation (RF-EMR) generator; 2, power amplifier; 3, horn antenna; 4, mice in the exposure group were placed in a home-made plastic box (35×24×24 cm) with vents on the surface. 5, a separate room with walls hanging microwave shielding materials.


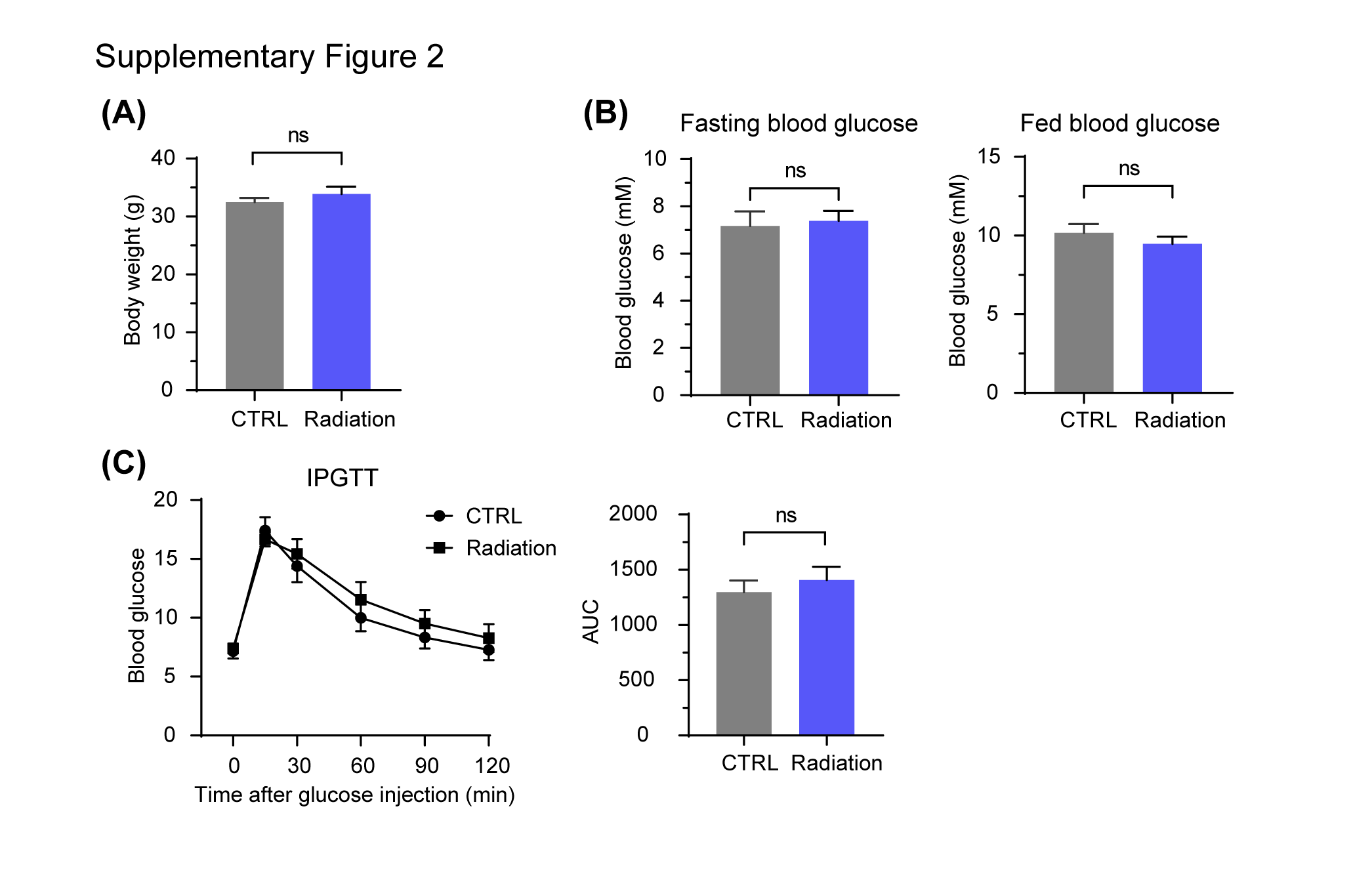


Supplementary Figure 2. Glucose metabolism of F0 after RF-EMR exposure. (A) The body weights of mice in the Radiation and CTRL group following 14-week RF-EMR radiation. (B) The levels of fasting blood glucose and fed glucose between the two group mice (after 14 weeks exposure). (C) The intraperitoneal glucose tolerance test (IPGTT) and the area under the curve (AUC) in 14-week RF-EMR radiation male mice. (A-C) CTRL, mice in sham group; Radiation, mice in RF-EMR exposure group. ns, not significant. n = 6 for each group. Student’s *t*-test.

## Supplementary Tables

**Supplementary Table 1. Primers used in this study**

| **Gene** | **Primer Sequences** | |
| --- | --- | --- |
|  | **Forward** | **Reverse** |
| Gck | GGCTTCACCTTCTCCTTCCC | TGTTGTTCCCTTCTGCTCCG |
| Pklr | GAGTCGGAGGTGGAAATTGT | GTCCACCCACACTGTCTTTG |
| G6pc | TCGGAGACTGGTTCAACCTC | TCACAGGTGACAGGGAACTG |
| Pck1 | CGCAAGCTGAAGAAATATGACA | GATGACTGTCTTGCTTTCGATC |
| Pygl | TGGCAGAAGTGGTGAACAATGAC | CCGTGGAGATCTGCTCCGATA |
| Foxo1 | GTGGATGGTGAAGAGCGTGC | AAGGGACAGATTGTGGCGAA |
| Irs1 | GCCAGAGGATCGTCAATAGC | AGACGTGAGGTCCTGGTTGT |
| Irs2  Actin | GGTCCAGGCACTGGAGCTTTG  GAAATCGTGCGTGACATCAAAG | GGGGCTGGTAGCGCTTCACT  TGTAGTTTCATGGATGCCACAG |
